# Association of Radioactive Iodine Treatment With Cancer Mortality in Patients With Hyperthyroidism

**DOI:** 10.1001/jamainternmed.2019.0981

**Published:** 2019-07-01

**Authors:** Cari M. Kitahara, Amy Berrington de Gonzalez, Andre Bouville, Aaron B. Brill, Michele M. Doody, Dunstana R. Melo, Steven L. Simon, Julie A. Sosa, Mark Tulchinsky, Daphnée Villoing, Dale L. Preston

**Affiliations:** 1 Radiation Epidemiology Branch, Division of Cancer Epidemiology and Genetics, National Cancer Institute, National Institutes of Health, Bethesda, Maryland; 2 Department of Radiology, Vanderbilt University Medical Center, Nashville, Tennessee; 3 Melohill Technology LLC, Rockville, Maryland; 4 Epidemiology and Biostatistics Program, Division of Cancer Epidemiology and Genetics, National Cancer Institute, National Institutes of Health, Bethesda, Maryland; 5 Department of Surgery, University of California, San Francisco, San Francisco; 6 Department of Radiology, Milton S. Hershey Medical Center, Pennsylvania State University, Hershey; 7 Hirosoft International, Eureka, California

## Abstract

**Importance:**

Radioactive iodine (RAI) has been used extensively to treat hyperthyroidism since the 1940s. Although widely considered a safe and effective therapy, RAI has been associated with elevated risks of total and site-specific cancer death among patients with hyperthyroidism.

**Objective:**

To determine whether greater organ- or tissue-absorbed doses from RAI treatment are associated with overall and site-specific cancer mortality in patients with hyperthyroidism.

**Design, Setting, and Participants:**

This cohort study is a 24-year extension of the multicenter Cooperative Thyrotoxicosis Therapy Follow-up Study, which has followed up US and UK patients diagnosed and treated for hyperthyroidism for nearly 7 decades, beginning in 1946. Patients were traced using records from the National Death Index, Social Security Administration, and other resources. After exclusions, 18 805 patients who were treated with RAI and had no history of cancer at the time of the first treatment were eligible for the current analysis. Excess relative risks (ERRs) per 100-mGy dose to the organ or tissue were calculated using multivariable-adjusted linear dose-response models and were converted to relative risks (RR = 1 + ERR). The current analyses were conducted from April 28, 2017, to January 30, 2019.

**Exposures:**

Mean total administered activity of sodium iodide I 131 was 375 MBq for patients with Graves disease and 653 MBq for patients with toxic nodular goiter. Mean organ or tissue dose estimates ranged from 20 to 99 mGy (colon or rectum, ovary, uterus, prostate, bladder, and brain/central nervous system), to 100 to 400 mGy (pancreas, kidney, liver, stomach, female breast, lung, oral mucosa, and marrow), to 1.6 Gy (esophagus), and to 130 Gy (thyroid gland).

**Main Outcomes and Measures:**

Site-specific and all solid-cancer mortality.

**Results:**

A total of 18 805 patients were included in the study cohort, and the mean (SD) entry age was 49 (14) years. Most patients were women (14 671 [78.0%]), and most had a Graves disease diagnosis (17 615 [93.7%]). Statistically significant positive associations were observed for all solid cancer mortality (n = 1984; RR at 100-mGy dose to the stomach = 1.06; 95% CI, 1.02-1.10; *P* = .002), including female breast cancer (n = 291; RR at 100-mGy dose to the breast = 1.12; 95% CI, 1.003-1.32; *P* = .04) and all other solid cancers combined (n = 1693; RR at 100-mGy dose to the stomach = 1.05; 95% CI, 1.01-1.10; *P* = .01). The 100-mGy dose to the stomach and breast corresponded to a mean (SD) administered activity of 243 (35) MBq and 266 (58) MBq in patients with Graves disease. For every 1000 patients with hyperthyroidism receiving typical doses to the stomach (150 to 250 mGy), an estimated lifetime excess of 19 (95% CI, 3-40) to 32 (95% CI, 5-66) solid cancer deaths could occur.

**Conclusions and Relevance:**

In RAI-treated patients with hyperthyroidism, greater organ-absorbed doses appeared to be modestly positively associated with risk of death from solid cancer, including breast cancer. Additional studies are needed of the risks and advantages of all major treatment options available to patients with hyperthyroidism.

## Key Points

**Question:**

Is radioactive iodine absorbed dose associated with overall and site-specific cancer mortality in patients with hyperthyroidism?

**Finding:**

In this cohort study of 18 805 patients with hyperthyroidism treated with radioactive iodine, a statistically significant positive dose-response relationship for risk of death was observed for all solid cancers (6% increase in risk per 100-mGy dose to the stomach), breast cancer (12% increase in risk per 100-mGy dose to the breast), and all solid cancers excluding breast (5% increase in risk per 100-mGy dose to the stomach).

**Meaning:**

This study’s findings suggest a modest positive association between greater organ-absorbed doses of radioactive iodine and risk of solid cancer death; additional studies are needed to fully weigh the risks and advantages of radioactive iodine and other treatment options for patients with hyperthyroidism.

## Teaser

This cohort study evaluates the level of ionizing radiation exposure and risk of cancer death from a common treatment for hyperthyroidism in a large and longest-running cohort of patients with hyperthyroidism.

## Introduction

In the United States, the prevalence of hyperthyroidism is 1.2% (0.5% overt and 0.7% subclinical), and most cases are due to Graves disease.^1^ Radioactive iodine (RAI; sodium iodide I 131, or Na^131^I) has been extensively used to treat hyperthyroidism since the 1940s and has been the preferred first-line treatment by US physicians for uncomplicated Graves disease.^2,3^ However, in recent decades, preference for RAI therapy as a primary treatment for Graves disease has declined in favor of antithyroid drugs, likely reflecting the increased awareness of an association between RAI and worsening of Graves ophthalmopathy as well as concerns about risks of radiation-induced cancer, whereas preference for initial surgical treatment has remained low.^3,4^

Postsurgical RAI ablation therapy for differentiated thyroid cancer (eg, >100 mCi) has been associated with an increased risk of secondary malignant neoplasms, including bone and soft-tissue sarcomas, salivary gland and digestive tract cancers, and leukemia.^5,6,7,8,9^ However, relatively few cohort studies have evaluated cancer risk after RAI for hyperthyroidism, which involves much lower administered activities (typically 10-15 mCi),^1^ and findings have been inconsistent.^2,10,11,12,13,14^ In previous studies, the amount of administered activity was used as a surrogate measure of radiation exposure^2,10,11,12,13^; however, radiation absorption varies by organ and can vary substantially across patients for a given administered dose. To date, risks of specific cancers have not been precisely quantified with well-substantiated estimates of absorbed dose to individual organs, which account for administered activity along with anatomic and physiologic patient characteristics associated with these effects.

The largest and most comprehensive study to date on this topic used data from Cooperative Thyrotoxicosis Therapy Follow-up Study, which had a cohort of more than 35 000 patients with hyperthyroidism (65% of whom were treated with RAI) enrolled between 1946 and 1964 in the United States and United Kingdom and with mortality follow-up through 1990.^2^ In that study, total and site-specific cancer mortality rates were not found to be elevated in RAI-treated patients compared with the general population. Although thyroid cancer mortality was elevated 4-fold in RAI-treated patients, this risk did not appear to be associated with radiation exposure from the treatment, given that greater administered activity, used as a proxy measure for absorbed dose to the thyroid, was not associated with thyroid cancer mortality after accounting for a 5-year latency period. Greater estimated red bone marrow–absorbed doses were not associated with death from hematopoietic and lymphoproliferative malignant neoplasms. Absorbed doses to other organs and tissues were calculated according to simplistic dosimetry assumptions but were not evaluated in relation to site-specific cancer death. On the basis of those findings, those authors concluded that RAI appeared to be a safe therapy for hyperthyroidism.^2^

We have since extended the follow-up of the Cooperative Thyrotoxicosis Therapy Follow-up Study by 24 years (maximum 68 years) and, we now use improved individual estimates of absorbed organ doses of I 131, as described in a previous publication.^15^ The objective of the current study was to evaluate the radiation dose-response relationships for site-specific cancer death among the RAI-treated patients with hyperthyroidism in the cohort.

## Methods

### Study Population

The mortality follow-up of the cohort is approved annually by the Institutional Review Board of the National Cancer Institute. Because the mortality follow-up is based on linkages with available databases and involves no direct contact with study participants, the requirement for informed consent was waived by the Institutional Review Board of the National Cancer Institute. The current analyses were conducted from April 28, 2017, to January 30, 2019.

The Cooperative Thyrotoxicosis Therapy Follow-up Study included all patients with a hyperthyroidism diagnosis between 1946 and 1964 at 25 US medical centers and 1 UK hospital.^16,17^ Comprehensive clinical data were abstracted from medical records. Patients were initially followed up through June 30, 1968. They were asked to return to the clinic at 2-year intervals for a physical examination, brief history, and additional blood studies. Follow-up information was obtained from the treating physicians or medical records, if available, or directly from the patients via mailed questionnaires. In 1984, investigators from the National Cancer Institute of the National Institutes of Health reassembled the cohort data from printed computer listings, microfiche, microfilm cassettes, and handwritten documents maintained at the collaborating medical centers.^2^ The records for 35 630 patients were compiled at 4 regional centers (Harvard University, Boston, Massachusetts; Memorial Sloan-Kettering Cancer Center, New York, New York; University of Southern California, Los Angeles; and Research Triangle Institute; Research Triangle Park, North Carolina). Patients were traced using records from the National Death Index, Social Security Administration, and other resources, and copies of death certificates were obtained and coded by trained nosologists. After excluding duplicates and incomplete records, the final data set included 35 593 patients, of whom 28 719 had complete mortality follow-up through 1990. Mortality follow-up continued for the US patients by linking with various tracing resources, including the Social Security Administration records to determine vital status and National Death Index Plus records to identify causes of death for decedents through December 31, 2014.

We further excluded those with no follow-up information, missing entry or exit dates, or exit dates that occurred on or before study entry (the first patient visit to a participating study clinic during the study enrollment period), resulting in 31 332 patients treated with RAI, surgical procedure, antithyroid drugs, or a combination of these options. From the 19 558 remaining patients who received RAI therapy, alone or in combination with other treatments, we excluded an additional 753 patients with a cancer diagnosis before study entry. After the exclusions, 18 805 patients were eligible for the analysis.

### Dose Estimation

A comprehensive dose assessment was conducted to estimate organ- or tissue-absorbed doses for cohort members who received RAI therapy (described in detail in a previous publication).^15^ In brief, an iodine mathematical biokinetic model was developed and calibrated using data from a kinetically well-characterized group of 197 patients with hyperthyroidism who had thyroid, blood, and urine measurements of I 131. The model estimates, along with individual patient data, were then applied to the full cohort of RAI-treated patients with hyperthyroidism and used to calculate the numbers of I 131 disintegrations in the source organs and tissues (regions with increased I 131 avidity). Iodine 131 photon and electron spectra from the International Commission on Radiological Protection^18^ were used to compute the S values^19^ (mean absorbed dose in a target region per unit disintegration of I 131 in a source region) on the adult reference voxel phantoms adopted by the International Commission on Radiological Protection^20^ for all important combinations of source and target regions. Absorbed dose to 26 target organs and tissues were calculated using new S value estimates^19^ for each patient and numbers of disintegrations in the source organs derived from the biokinetic model (from both diagnostic tests and therapeutic treatments).

### Statistical Analysis

For the current analyses, person-years at risk for each patient were computed from 5 years after the date of the last RAI treatment (if a typical latency period were assumed between radiation exposure and solid cancer occurrence) until the date of death, date last known to be alive for patients lost to follow-up, or end of follow-up (December 31, 2014). Dose-response analyses were conducted among the patients receiving RAI treatment by fitting multivariable linear excess relative risk (ERR) models to disease rates. To directly compare the strength of the radiation dose-response relationship across the specific causes of cancer death, we calculated the ERRs per 100 mGy absorbed dose to the target organ or tissue for that cancer site (with stomach dose as a whole-body dose surrogate) and computed 95% likelihood-based CIs around these estimates. These models have the following form:

*background(a, s, b, x)*[1+βd*f(y)],

in which the rate is a function of age(*a*), sex(*s*), birth cohort(*b*), other risk factors(*x*), and dose(*d*), and f(*y*) describes the effects of modifying factors (eg, attained age). Background rates included sex-dependent functions of attained age and birth cohort (continuous), with further adjustment for the following potential confounders, chosen a priori: Graves disease diagnosis (yes or no), additional surgical procedure (yes or no), and additional antithyroid drug treatment (yes or no).

Relative risks (RRs) at 100 mGy were calculated by adding 1 to these estimates (RR = 1 + ERR). Baseline clinical impression (eg, mild, moderate, severe hyperthyroidism, or unknown), as recorded by physicians in each center, was not retained in the final models because inclusion of this term did not statistically significantly improve the model fit or affect the estimates. Beginning follow-up 2 years, as opposed to 5 years, after the last treatment did not change the results for non–solid cancer mortality (leukemia excluding chronic lymphocytic leukemia and non-Hodgkin lymphoma). Stratified person-year computations and risk estimations were performed using Epicure, version 2.00.02 (Risk Sciences International).^21^ Two-sided hypothesis tests and 95% CIs were based on likelihood ratio tests, with a significance threshold of *P* = .05.

These risk estimates, along with baseline age-specific total and cause-specific death rates obtained from the 2014 Surveillance, Epidemiology, and End Results mortality data,^22^ were used to estimate the number of future cancer deaths estimated to be attributed to radiation exposure on the basis of a hypothetical population of patients who received the same organ or tissue dose at the same age (eg, 40 years) and in the same calendar year (eg, 2014). For this analysis, we used the risk of exposure-induced death method, which is the sum of the expected number of exposure-associated deaths at each age, taking into account competing risks.^23^ Estimates of the number of future cancer deaths were given per 1000 patients.

## Results

Of the 18 805 patients in the study cohort, 14 671 (78.0%) were women and 17 615 (93.7%) had Graves disease (Table 1). The mean (SD) age at entry was 49 (14) years.

**Table 1.** Selected Characteristics of the Study Population

Characteristic	Participants, No. (%)
Total	18 805 (100)
Age at study entry, y	
<30	1733 (9.2)
30-39	3055 (16.2)
40-49	4816 (25.6)
≥50	9201 (48.9)
Sex	
Male	4134 (22.0)
Female	14 671 (78.0)
Hyperthyroidism diagnosis	
Graves disease	17 615 (93.7)
Toxic nodular goiter	934 (5.0)
Unknown	256 (1.4)
Treatment combination	
RAI only	7182 (38.2)
RAI and surgical procedure	694 (3.7)
RAI and drugs	8675 (46.1)
RAI, surgical procedure, and drugs	2254 (12.0)
Vital status	
Alive or lost to follow-up	3321 (17.7)
Deceased	15 484 (82.3)
Study site	
Mount Sinai Hospital (New York, NY)	3042 (16.2)
Mayo Clinic (Rochester, MN)	1907 (10.1)
Massachusetts General (Boston, MA)	1726 (9.2)
Sheffield Hospital (Sheffield, UK)	1378 (7.3)
Columbia Presbyterian Hospital (New York, NY)	1190 (6.3)
Cedars Sinai Medical Center (Los Angeles, CA)	1093 (5.8)
Los Angeles County Hospitals (Los Angeles, CA)	1065 (5.7)
University of Michigan (Ann Arbor, MI)	978 (5.2)
University of Maryland (Baltimore, MD)	899 (4.8)
University of California (San Francisco, CA)	747 (4.0)
Beth Israel Hospital (Boston, MA)	678 (3.6)
University Hospitals of Cleveland (Cleveland, OH)	630 (3.4)
New York Hospital-Cornell (New York, NY)	612 (3.3)
Lahey Clinic (Boston, MA)	589 (3.1)
Montefiore Med Center (New York, NY)	560 (3.0)
White Memorial Hospital (Los Angeles, CA)	518 (2.8)
University of Cincinnati (Cincinnati, OH)	492 (2.6)
Memorial Sloan-Kettering (New York, NY)	283 (1.5)
Cleveland Metropolitan General Hospital (Cleveland, OH)	198 (1.1)
Strong Memorial Hospital (Rochester, NY)	125 (0.7)
St Louis University (St Louis, MO)	52 (0.3)
Ochsner Clinic (New Orleans, LA)	43 (0.2)

Abbreviation: RAI, radioactive iodine.

The treatment combination of RAI and antithyroid drugs was the most common (8675 [46.1%]), followed by RAI alone (7182 [38.2%]); RAI, surgical procedure, and drugs (2254 [12.0%]); and RAI and surgical procedure (694 [3.7%]). By number of RAI treatments, 12 387 patients (65.9%) received 1 treatment, 3629 (19.3%) received 2 treatments, 1317 (7.0%) received 3 treatments, and 1471 (7.8%) received more than 3 treatments. Mean (SD) total administered activity (including diagnostic and therapeutic activity) was 375 MBq (10.1 mCi) for patients with Graves disease (median [interquartile range (IQR)], 269 [187-419] MBq) and 653 MBq (17.6 mCi) for patients with toxic nodular goiter (median [IQR], 488 [301-792] MBq).

The highest mean estimated absorbed doses were to the thyroid (130 Gy) (to convert gray to rad, multiply by 100), followed by the esophagus (1.6 Gy); liver, oral mucosa, lung, stomach, red bone marrow, female breast, pancreas, kidney (100-400 mGy); and uterus, brain, bladder, ovary, prostate, and colon or rectum (20-99 mGy). Factors associated with total administered activity included number of RAI treatments, older age at entry, clinical impression of disease severity, toxic nodular goiter diagnosis, previous weight loss, history of heart disease and diabetes, and additional treatment with both surgical procedure and antithyroid drugs (Table 2).

**Table 2.** Baseline Demographic and Clinical Characteristics of Patients With Hyperthyroidism Treated With Radioactive Iodine

Baseline Characteristics	Tertile of Total Administered Activity, MBq		
	1 (n = 6296)	2 (n = 6248)	3 (n = 6261)
Total administered activity, mean (SD), MBq^a^	160 (45)	289 (46)	724 (500)
No. of RAI treatments, mean (SD)	1.3 (1.2)	1.4 (0.9)	2.4 (1.9)
Dose, mGy			
Stomach	65 (20)	120 (25)	320 (240)
Breast^b^	60 (21)	110 (29)	280 (210)
Thyroid	65 000 (31 000)	110 000 (41 000)	210 000 (160 000)
Study entry age, mean (SD), y	47 (13)	49 (14)	52 (14)
Sex, No. (%)			
Male	1288 (20.5)	1383 (22.1)	1463 (23.4)
Female	5008 (79.5)	4865 (77.9)	4798 (76.6)
Clinical impression, No. (%)			
Suspect	111 (1.8)	96 (1.5)	72 (1.2)
Mild	1758 (27.9)	1649 (26.4)	1567 (25.0)
Moderate	2564 (40.7)	2605 (41.7)	2639 (42.2)
Severe	439 (7.0)	596 (9.5)	857 (13.7)
Unknown	1424 (22.6)	1301 (20.8)	1126 (18.0)
Type of hyperthyroidism, No. (%)			
Graves disease	6127 (97.3)	5967 (95.5)	5521 (88.2)
Toxic nodular goiter	115 (1.8)	207 (3.3)	612 (9.8)
Intermediate status	54 (0.9)	74 (1.2)	128 (2.0)
Weight loss prior to surgical procedure, No. (%)	4165 (66.2)	3995 (63.9)	3911 (62.5)
Amount of weight loss prior to surgical procedure, mean (SD), lb	19 (13)	21 (14)	23 (16)
Medical history (prior to surgical procedure), No. (%)			
Coronary heart disease	257 (4.1)	442 (7.1)	654 (10.5)
Hypertensive heart disease	364 (5.8)	564 (9.0)	720 (11.5)
Rheumatic heart disease	137 (2.2)	191 (3.1)	222 (3.6)
Other heart disease	274 (4.4)	398 (6.4)	718 (11.5)
Diabetes	238 (3.8)	303 (4.9)	433 (6.9)
Treatment combinations, No. (%)			
RAI only	2589 (41.1)	2597 (41.6)	1996 (31.9)
RAI and surgical procedure	278 (4.4)	225 (3.6)	191 (3.1)
RAI and drugs	798 (12.5)	726 (11.6)	739 (11.8)
RAI, surgical procedure, and drugs	2640 (41.9)	2700 (43.2)	3335 (53.3)

Abbreviations: MBq, megabecquerel; RAI, radioactive iodine.

SI conversion factors: To convert pounds to kg, multiply by 0.45.

^a^ Includes diagnostic and therapeutic doses.

^b^ Women only.

During follow-up (mean of 26 years; maximum of 68 years), 15 484 deaths (82.3%) were recorded. Malignancy was the primary cause for 2366 deaths (15.3%). After excluding deaths in the first 5 years after the last RAI treatment, positive dose-response relationships were observed for mortality from most of the individual solid cancers evaluated (Table 3), with statistically significant associations observed for female breast cancer (n = 291; RR at 100-mGy dose to the breast = 1.12; 95% CI, 1.003-1.32; *P* = .04) and all other solid cancers combined (RR at 100-mGy dose to the stomach = 1.05; 95% CI, 1.01-1.10; *P* = .01). For all solid cancer mortality (n = 1984), the RR at 100-mGy dose to the stomach was 1.06 (95% CI, 1.02-1.10; *P* = .002). The 100-mGy dose to the stomach corresponded to a mean (SD) administered activity of 243 (35) MBq in patients with Graves disease (median [IQR], 234 [223-263] MBq), whereas the 100-mGy dose to the breast corresponded to a mean (SD) administered activity of 266 (58) MBq (median [IQR], 260 [224-297] MBq). The RR for thyroid cancer mortality was evaluated at 100 Gy thyroid-absorbed dose, a typical high dose received by the thyroid gland; however, the variability around this estimate was wide and based on only 15 deaths (RR = 1.20; 95% CI, <1.00 to 610). Because these estimates were based on linear dose-response models, risks associated with higher (or lower) doses can be directly estimated from these models.

**Table 3.** Relative Risks and 95% CIs for Cancer-Specific Mortality Among Patients With Hyperthyroidism Treated With Radioactive Iodine

Cause of Cancer Death^a^	Absorbed Dose, mGy		Dose-Response Relationship			Cause-Specific Cancer Death Attributed to Irradiation, No. (%)^c^
	Target Organ or Tissue	Organ- or Tissue-Absorbed Dose, Mean (SD)	No. of Deaths	At 100-mGy Organ- or Tissue-Absorbed Dose, RR (95% CI)^b^	*P* Value	
Solid cancers						
Oral cavity	Mucosa	320 (320)	31	0.99 (<0.99-1.30)	>.50	
Esophageal	Esophagus	1600 (1500)	38	1.01 (<1.00-1.87)	>.50	
Stomach	Stomach	170 (180)	97	1.03 (<0.98-1.28)	>.50	
Colon	Colon	23 (25)	258	1.19 (<0.80-2.17)	>.50	
Rectal	Rectum	18 (19)	49	1.54 (<0.75-6.53)	>.50	
Liver	Liver	390 (460)	34	0.99 (<0.99-1.12)	>.50	
Pancreatic	Pancreas	110 (120)	132	1.13 (<0.97-1.56)	.27	
Lung or bronchus	Lung	310 (310)	437	1.02 (<0.99-1.07)	.31	
Bladder	Bladder	49 (50)	54	0.96 (<0.96-2.15)	>.50	
Kidney	Kidney	110 (130)	48	1.32 (<0.97-9.34)	>.50	
Brain or central nervous system	Brain	58 (56)	39	1.07 (<0.93-2.98)	>.50	
Thyroid	Thyroid	130 000 (110 000)	15	1.20 (<1.00-6.10)^d^	>.50	
Female breast	Breast	150 (160)	291	1.12 (1.00-1.32)	.04	41.9 (14)
Uterine	Uterus	63 (69)	63	1.54 (0.98-3.42)	.07	
Ovarian	Ovary	38 (42)	104	1.32 (<0.90-2.46)	.30	
Prostate	Prostate	42 (41)	52	1.04 (<0.86-2.42)	>.50	
All other solid cancers	Stomach	170 (180)	242	1.02 (<0.98-1.16)	>.50	
Leukemia (excluding CLL)	Marrow	160 (160)	59	0.97 (<0.96-1.26)	>.50	
Non-Hodgkin lymphoma	Marrow	160 (160)	70	1.07 (<0.96-1.54)	>.50	
Multiple myeloma	Marrow	160 (160)	30	1.69 (<0.97->6.00)	>.50	
All solid cancers combined	Stomach	170 (180)	1984	1.06 (1.02-1.10)	.002	154.7 (8)
All solid cancers excluding female breast	Stomach	170 (180)	1693	1.05 (1.01-1.10)	.01	117.2 (7)

Abbreviations: CLL, chronic lymphocytic leukemia; ERR, excess relative risk; Gy, gray; RAI, radioactive iodine; RR, relative risk.

^a^ Patient deaths that occurred in the first 5 years after last RAI treatment were excluded.

^b^ Based on a linear excess RR model using continuous values of organ- or tissue-absorbed dose (per 100 mGy), in which RR = 1 + ERR. Background rates include terms for sex, sex-specific attained age and birth cohort patterns, Graves disease diagnosis (yes or no), additional treatment with surgical procedure (yes or no), and additional treatment with antithyroid drugs (yes or no). To calculate RRs at 100*x mGy organ or tissue dose, use the following equation: [(ERR)*x +1].

^c^ Estimates are shown only when the corresponding RRs were statistically significant at *P* < .05.

^d^ RR at 100 Gy.

The dose-response relationships for all solid cancer, breast cancer, and all solid cancer (excluding breast) did not change after restricting to patients receiving a single RAI treatment or RAI-only treatment (no surgical procedure or antithyroid drugs) (eTable 1 in the Supplement) or after excluding patients with toxic nodular goiter. The shapes of the dose-response relationships were consistent with linearity (Figure). The RRs for all solid cancer mortality (but not for breast cancer or all other solid cancer) decreased with increasing attained age. We observed no evidence of a dose-response relationship for mortality from leukemia, excluding chronic lymphocytic leukemia (59 deaths; RR at 100 mGy = 0.97; 95% CI, <0.96 to 1.26), non-Hodgkin lymphoma (70 deaths; RR at 100 mGy = 1.07; 95% CI, <0.96 to 1.54), or multiple myeloma (30 deaths; RR at 100 mGy = 1.69; 95% CI, <0.97 to >6.0) (Table 3). We estimated that 8% of the solid cancer deaths, including 14% of breast cancer and 7% of all other solid cancer deaths, during follow-up were attributed to radiation in these patients.

**Figure.**
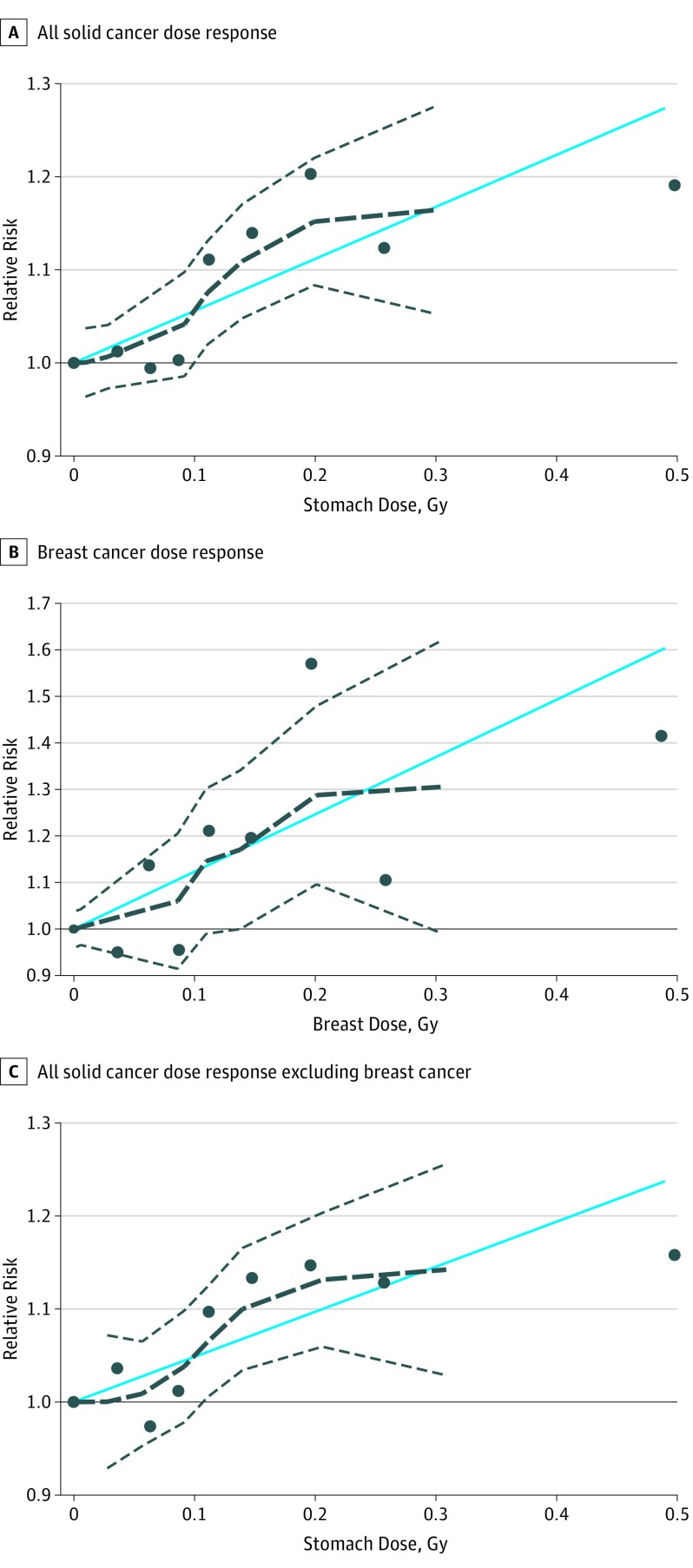
Relative Risks for Solid Cancer, Female Breast Cancer, and Solid Cancer Without Female Breast Cancer Mortality Among Patients With Hyperthyroidism Patients were cancer free at the time of radioactive iodine (RAI) treatment. Relative risks were compared across organ- or tissue-absorbed dose (in grays [to convert to the conventional unit rad, multiply by 100]). Solid horizontal lines represent the relative risk reference value (1). Solid blue lines represent the estimated log-linear dose-response relationships. Dashed black lines represent the smoothed dose-response relationships, and dashed gray lines represent 95% CIs. Black dots represent the relative risk at each organ dose category. Background rates include terms for sex, sex-specific attained age and birth cohort patterns, Graves disease diagnosis, additional treatment with surgical procedure, and additional treatment with antithyroid drugs.

Combining these RR estimates with current US mortality rates, we estimated that 13 (95% CI, 2-27) excess solid cancer deaths, including 3 breast cancer deaths (95% CI, 0.1-7), would occur for every 1000 patients (80% women), receiving 100 mGy absorbed dose to the stomach or breast at age 40 years. For every 1000 patients currently treated at age 50 years, we estimated an excess of 12 (95% CI, 2-26) radiation-associated solid cancer deaths, including 3 breast cancer deaths (95% CI, 0.1-7) (eTable 2 in the Supplement). Of the excess solid cancer deaths, only 8% in the age 40 years group and 25% in the age 50 years group would be expected to occur in the first 20 years after treatment. Because higher administered activities are now currently recommended for the treatment of patients with Graves disease (370-555 MBq),^1^ we also calculated excess solid cancer deaths at 150-mGy, 200-mGy, and 250-mGy dose to the stomach, which would be more typical of current treatment practices (eTable 2 in the Supplement). At these dose levels, we would expect between 19 and 32 excess solid cancer deaths per 1000 patients treated at age 40 years and between 18 and 31 excess solid cancer deaths per 1000 patients treated at age 50 years.

## Discussion

Using data from the world’s largest cohort, to date, of RAI-treated patients with hyperthyroidism, we investigated the association between organ- or tissue-absorbed dose and site-specific cancer death. To our knowledge, this is the first study to characterize the dose-response relationship between RAI treatment and site-specific cancer mortality in patients with hyperthyroidism using reliable estimates of absorbed dose to exposed organs or tissues. We observed that the RR of death from solid cancer, including breast cancer and all other solid cancers combined, increased with greater doses to organs or tissues. We estimated that RAI treatment could result in a small long-term increase in the expected number of solid cancer deaths associated with radiation exposure among patients with hyperthyroidism.

Smaller previously studied European cohorts showed evidence of an increased risk of total and/or site-specific cancers following RAI treatment, including cancers in organs that took up radioiodine or were exposed along the I 131 distribution pathways (eg, salivary and digestive organs).^2,10,11,12,13,14^ However, results from these earlier studies were inconsistent, and it remains unclear, particularly in studies relying on an external (eg, general population) comparison group,^24^ whether those findings represent consequences from the treatment or the underlying disease,^25,26^ or if they were biased owing to confounding. Confounding by indication, which occurs when the reasons for choosing a specific treatment option are associated with the outcome under study,^27,28^ is of particular concern in observational studies that compare cancer or other disease risks by treatment type, which was the main approach used in the previous analysis of this cohort.^2^ By focusing the present analysis on patients treated with RAI, we were able to minimize this bias.

To our knowledge, this study is the first to provide direct evidence of an association between internal exposure from I 131 and breast cancer risk. A similar study of a small cohort of RAI-treated patients with hyperthyroidism in Finland found an elevated risk for breast cancer; however, the dose-response relationship was not evaluated in that study.^11^ Ecological data from the Chernobyl radiation accident in Russia previously suggested such an association, with a 2-fold higher breast cancer risk in the most (vs least) contaminated districts starting approximately 10 years after the accident.^29^ The dose-response estimate for breast cancer mortality in the present study (RR at 100 mGy = 1.12; 95% CI, 1.003-1.32) was consistent with similar estimates from other populations exposed to ionizing radiation in adulthood, including the Life Span Study of atomic bomb survivors in Japan (RR at 100 mGy at age 30 years with attained age of 70 years = 1.09; 95% CI, 1.06-1.13)^30^; the US Radiologic Technologists Study (RR at 100 mGy = 1.07; 95% CI, 0.99-1.19)^31^; and smaller environmentally, occupationally, and medically exposed populations.^32,33,34^ This consistency lends credibility to the estimated doses and findings reported in this study.

Despite the high thyroid doses received (mean, 130 Gy), we found no evidence of a dose-response relationship with thyroid cancer death. Previous studies have found that children undergoing high-dose radiotherapy for a first primary cancer were at an increased risk of developing secondary thyroid cancer, but the risk was attenuated at higher doses (>30 Gy), presumably because of cell killing.^35^ Whether malignant transformation of residual thyroid cells is possible at the higher thyroid doses received by patients with hyperthyroidism, who are generally exposed in adulthood when the thyroid gland is less susceptible to radiation exposure, remains unclear. The ability to evaluate dose-response relationships for cancers that are rarely fatal, such as thyroid cancer, in this study was limited as the cohort was followed for mortality outcomes only. Studies with high-quality dosimetry and cancer incidence follow-up are needed to better characterize the risk of thyroid cancer in adults and children after RAI treatment for hyperthyroidism.

The lack of evidence of a dose-response for leukemia mortality in the current study was somewhat unexpected, given that an elevated risk of leukemia was observed in patients with thyroid cancer who received much higher levels of administered activity.^5,6,7,8,9^ Low-dose radiation exposure was also found to be associated with leukemia risk in previous studies.^36,37^ However, greater uncertainty in the calculation of red bone marrow activity compared with that of other organ- or tissue-absorbed doses,^15^ combined with the low-dose range of exposure and relatively small number of leukemia deaths, limited our ability to detect a dose-response relationship in this setting. Thus, we cannot rule out an association between radiation treatment for hyperthyroidism and leukemia induction.

### Strengths and Limitations

Strengths of this study included the large cohort size, long-term follow-up, detailed clinical information, and analytic methods to minimize confounding. In addition, we used high-quality, individualized organ- and tissue-specific dose estimates derived from a biokinetic model that was developed and calibrated in a small group of well-characterized patients with hyperthyroidism, with individual radioactivity measurements, calculated S values, and clinical measurements available for all (administered activity) or most (thyroid mass and percentage uptake) of the RAI-treated patients in the full study.^15^

Nonetheless, major uncertainties in the organ (particularly non-thyroid) dose estimations may have biased the study findings toward the null. The small number of deaths for many cancers of interest, together with relatively small doses to organs other than the thyroid, limited our power to detect statistically significant associations for those outcomes. Considering the number of statistical tests performed, some results may be because of chance; therefore, the results should be interpreted with caution. As the results were unadjusted for smoking, obesity, alcohol use, or reproductive factors, confounding by these known cancer risk factors is possible. Although the results were adjusted for additional treatment with antithyroid drugs (yes or no), residual confounding may be possible, as this information may not have been complete for all patients, and the information on type, quantity, and dates of use was not recorded in the database.^2^ Given that the types of antithyroid drugs administered to patients in the cohort differed from those more commonly prescribed in recent years (eg, methimazole and carbimazole),^2,38^ additional studies that can more precisely evaluate the long-term health implications of current antithyroid drug therapies, including in the context of RAI therapy.^39^ Confounding by severity of the underlying hyperthyroid status and other concomitant diseases was a potential source of bias, but we found that the results changed little after adjusting for differences in clinical impression of disease severity.

## Conclusions

Hyperthyroid treatment decisions should take into consideration the balance of risks with advantages of each available treatment option as well as patient preference, health status, and access to these options. We believe the results of this study provide quantitative estimates of the risks of radiation-associated cancer deaths in RAI-treated patients with hyperthyroidism, which were previously not well understood, and suggest that the risk of death from solid cancer (including breast cancer) increases with the greater absorbed dose to exposed organs and tissues. Additional studies are needed to fully weigh the risks and advantages of RAI and other major treatment options available to patients with hyperthyroidism.

## Notes

Supplement. **eTable 1.** Relative Risks (RRs) and 95% Confidence Intervals (CIs) for Cancer-Specific Mortality at 100 mGy Organ/Tissue Absorbed Dose Among Hyperthyroid Patients Treated With RAI **eTable 2.** Estimates of Radiation-Associated Excess Solid Cancer Deaths (95% CIs) per 1,000 Hyperthyroidism Patients (80% Women) treated With Radioactive Iodine, by Age at treatment and Estimated Stomach Dose

## References

[1] Ross DS, Burch HB, Cooper DS, 2016 American Thyroid Association guidelines for diagnosis and management of hyperthyroidism and other causes of thyrotoxicosis. Thyroid. 2016;26(10):1343-1421. doi:10.1089/thy.2016.0229 27521067

[2] Ron E, Doody MM, Becker DV, ; Cooperative Thyrotoxicosis Therapy Follow-up Study Group Cancer mortality following treatment for adult hyperthyroidism. JAMA. 1998;280(4):347-355. doi:10.1001/jama.280.4.347 9686552

[3] Burch HB, Burman KD, Cooper DS A 2011 survey of clinical practice patterns in the management of Graves’ disease. J Clin Endocrinol Metab. 2012;97(12):4549-4558. doi:10.1210/jc.2012-2802 23043191

[4] Bartalena L, Burch HB, Burman KD, Kahaly GJA A 2013 European survey of clinical practice patterns in the management of Graves’ disease. Clin Endocrinol (Oxf). 2016;84(1):115-120. doi:10.1111/cen.12688 25581877

[5] Teng CJ, Hu YW, Chen SC, Use of radioactive iodine for thyroid cancer and risk of second primary malignancy: a nationwide population-based study. J Natl Cancer Inst. 2015;108(2):djv314. doi:10.1093/jnci/djv314 26538627

[6] Teepen JC, Curtis RE, Dores GM, Risk of subsequent myeloid neoplasms after radiotherapy treatment for a solid cancer among adults in the United States, 2000-2014. Leukemia. 2018;32(12):2580-2589. doi:10.1038/s41375-018-0149-2 29795414

[7] Molenaar RJ, Sidana S, Radivoyevitch T, Risk of hematologic malignancies after radioiodine treatment of well-differentiated thyroid cancer. J Clin Oncol. 2018;36(18):1831-1839. doi:10.1200/JCO.2017.75.0232 29252123 PMC8462524

[8] Kim C, Bi X, Pan D, The risk of second cancers after diagnosis of primary thyroid cancer is elevated in thyroid microcarcinomas. Thyroid. 2013;23(5):575-582. doi:10.1089/thy.2011.0406 23237308 PMC3643257

[9] Yu CY, Saeed O, Goldberg AS, A systematic review and meta-analysis of subsequent malignant neoplasm risk after radioactive iodine treatment of thyroid cancer [published online November 27, 2018]. Thyroid. 30370820 10.1089/thy.2018.0244

[10] Holm LE, Hall P, Wiklund K, Cancer risk after iodine-131 therapy for hyperthyroidism. J Natl Cancer Inst. 1991;83(15):1072-1077. doi:10.1093/jnci/83.15.1072 1875414

[11] Metso S, Auvinen A, Huhtala H, Salmi J, Oksala H, Jaatinen P Increased cancer incidence after radioiodine treatment for hyperthyroidism. Cancer. 2007;109(10):1972-1979. doi:10.1002/cncr.22635 17393376

[12] Hall P, Berg G, Bjelkengren G, Cancer mortality after iodine-131 therapy for hyperthyroidism. Int J Cancer. 1992;50(6):886-890. doi:10.1002/ijc.2910500611 1555888

[13] Franklyn JA, Maisonneuve P, Sheppard M, Betteridge J, Boyle P Cancer incidence and mortality after radioiodine treatment for hyperthyroidism: a population-based cohort study. Lancet. 1999;353(9170):2111-2115. doi:10.1016/S0140-6736(98)12295-X 10382695

[14] Ryödi E, Metso S, Jaatinen P, Cancer incidence and mortality in patients treated either with RAI or thyroidectomy for hyperthyroidism. J Clin Endocrinol Metab. 2015;100(10):3710-3717. doi:10.1210/jc.2015-1874 26262435

[15] Melo DR, Brill AB, Zanzonico P, Organ dose estimates for hyperthyroid patients treated with (131)I: an update of the thyrotoxicosis follow-up study. Radiat Res. 2015;184(6):595-610. doi:10.1667/RR14160.1 26579944

[16] Saenger EL, Thoma GE, Tompkins EA Incidence of leukemia following treatment of hyperthyroidism. Preliminary report of the Cooperative Thyrotoxicosis Therapy Follow-Up Study. JAMA. 1968;205(12):855-862. doi:10.1001/jama.1968.03140380059014 5695509

[17] Dobyns BM, Sheline GE, Workman JB, Tompkins EA, McConahey WM, Becker DV Malignant and benign neoplasms of the thyroid in patients treated for hyperthyroidism: a report of the cooperative thyrotoxicosis therapy follow-up study. J Clin Endocrinol Metab. 1974;38(6):976-998. doi:10.1210/jcem-38-6-976 4134013

[18] Eckerman K, Endo A ICRP Publication 107. Nuclear decay data for dosimetric calculations. Ann ICRP. 2008;38(3):7-96. 19285593 10.1016/j.icrp.2008.10.004

[19] Lamart S, Simon SL, Bouville A, Moroz BE, Lee C S values for 131I based on the ICRP adult voxel phantoms. Radiat Prot Dosimetry. 2016;168(1):92-110. doi:10.1093/rpd/ncv016 25829162 PMC4729327

[20] Menzel HG, Clement C, DeLuca P ICRP Publication 110. Realistic reference phantoms: an ICRP/ICRU joint effort. A report of adult reference computational phantoms. Ann ICRP. 2009;39(2):1-164. 19897132 10.1016/j.icrp.2009.09.001

[21] Preston DL, Lubin JH, Pierce DA, McConney ME, Shilnikova NS EPICURE user manual and command summary. http://epicurehelp.risksciences.com. Accessed February 20, 2018.

[22] Surveillance, Epidemiology, and End Results Program US mortality data, 1969-2015. National Cancer Institute. https://seer.cancer.gov/mortality/. Accessed February 20, 2018.

[23] Thomas D, Darby S, Fagnani F, Hubert P, Vaeth M, Weiss K Definition and estimation of lifetime detriment from radiation exposures: principles and methods. Health Phys. 1992;63(3):259-272. doi:10.1097/00004032-199209000-00001 1644562

[24] Kahlert J, Gribsholt SB, Gammelager H, Dekkers OM, Luta G Control of confounding in the analysis phase: an overview for clinicians. Clin Epidemiol. 2017;9:195-204. doi:10.2147/CLEP.S129886 28408854 PMC5384727

[25] Journy NMY, Bernier MO, Doody MM, Alexander BH, Linet MS, Kitahara CM Hyperthyroidism, hypothyroidism, and cause-specific mortality in a large cohort of women. Thyroid. 2017;27(8):1001-1010. doi:10.1089/thy.2017.0063 28578598 PMC5564026

[26] Søgaard M, Farkas DK, Ehrenstein V, Jørgensen JO, Dekkers OM, Sørensen HT Hypothyroidism and hyperthyroidism and breast cancer risk: a nationwide cohort study. Eur J Endocrinol. 2016;174(4):409-414. doi:10.1530/EJE-15-0989 26863886

[27] Kyriacou DN, Lewis RJ Confounding by indication in clinical research. JAMA. 2016;316(17):1818-1819. doi:10.1001/jama.2016.16435 27802529

[28] Walker AM Confounding by indication. Epidemiology. 1996;7(4):335-336. 8793355

[29] Pukkala E, Kesminiene A, Poliakov S, Breast cancer in Belarus and Ukraine after the Chernobyl accident. Int J Cancer. 2006;119(3):651-658. doi:10.1002/ijc.21885 16506213

[30] Preston DL, Ron E, Tokuoka S, Solid cancer incidence in atomic bomb survivors: 1958-1998. Radiat Res. 2007;168(1):1-64. doi:10.1667/RR0763.1 17722996

[31] Preston DL, Kitahara CM, Freedman DM, Breast cancer risk and protracted low-to-moderate dose occupational radiation exposure in the US Radiologic Technologists Cohort, 1983-2008. Br J Cancer. 2016;115(9):1105-1112. doi:10.1038/bjc.2016.292 27623235 PMC5117787

[32] Preston DL, Mattsson A, Holmberg E, Shore R, Hildreth NG, Boice JD Jr Radiation effects on breast cancer risk: a pooled analysis of eight cohorts. Radiat Res. 2002;158(2):220-235. doi:10.1667/0033-7587(2002)158[0220:REOBCR]2.0.CO;2 12105993

[33] Davis FG, Yu KL, Preston D, Epifanova S, Degteva M, Akleyev AV Solid cancer incidence in the Techa River Incidence cohort: 1956-2007. Radiat Res. 2015;184(1):56-65. doi:10.1667/RR14023.1 26121228

[34] Sokolnikov M, Preston D, Gilbert E, Schonfeld S, Koshurnikova N Radiation effects on mortality from solid cancers other than lung, liver, and bone cancer in the Mayak worker cohort: 1948-2008. PLoS One. 2015;10(2):e0117784. doi:10.1371/journal.pone.0117784 25719381 PMC4342229

[35] Veiga LH, Holmberg E, Anderson H, Thyroid cancer after childhood exposure to external radiation: an updated pooled analysis of 12 studies. Radiat Res. 2016;185(5):473-484. doi:10.1667/RR14213.1 27128740 PMC4893786

[36] Leuraud K, Richardson DB, Cardis E, Ionising radiation and risk of death from leukaemia and lymphoma in radiation-monitored workers (INWORKS): an international cohort study. Lancet Haematol. 2015;2(7):e276-e281. doi:10.1016/S2352-3026(15)00094-0 26436129 PMC4587986

[37] Little MP, Wakeford R, Borrego D, Leukaemia and myeloid malignancy among people exposed to low doses (<100 mSv) of ionising radiation during childhood: a pooled analysis of nine historical cohort studies. Lancet Haematol. 2018;5(8):e346-e358. doi:10.1016/S2352-3026(18)30092-9 30026010 PMC6130888

[38] Burch HB, Cooper DS Management of Graves disease: a review. JAMA. 2015;314(23):2544-2554. doi:10.1001/jama.2015.16535 26670972

[39] Walter MA, Briel M, Christ-Crain M, Effects of antithyroid drugs on radioiodine treatment: systematic review and meta-analysis of randomised controlled trials. BMJ. 2007;334(7592):514. doi:10.1136/bmj.39114.670150.BE 17309884 PMC1819480

