# Supplementary material for: Association of Radioactive Iodine Treatment With Cancer Mortality in Patients With Hyperthyroidism
Source: JAMA Intern Med. 2019 Jul 1;179(8):1034–42. doi: 10.1001/jamainternmed.2019.0981 (PMC6604114; doi:10.1001/jamainternmed.2019.0981)
Supplement: Supplement. — eTable 1. Relative Risks (RRs) and 95% Confidence Intervals (CIs) for Cancer-Specific Mortality at 100 mGy Organ/Tissue Absorbed Dose Among Hyperthyroid Patients Treated With RAI eTable 2. Estimates of Radiation-Associated Excess Solid Cancer Deaths (95% CIs) per 1,000 Hyperthyroidism Patients (80% Women) treated With Radioactive Iodine, by Age at treatment and Estimated Stomach Dose [file jamainternmed-179-1034-s001.pdf]

Supplementary Online Content

Kitahara CM, Berrington de Gonzalez A, Bouville A, et al. Association of radioactive iodine treatment with cancer mortality in patients with hyperthyroidism. *JAMA Intern Med.* Published online July 1, 2019. doi:10.1001/jamainternmed.2019.0981

eTable 1. Relative Risks (RRs) and 95% Confidence Intervals (CIs) for Cancer-Specific Mortality at 100 mGy Organ/Tissue Absorbed Dose Among Hyperthyroid Patients Treated With RAI

eTable 2. Estimates of Radiation-Associated Excess Solid Cancer Deaths (95% CIs) per 1,000 Hyperthyroidism Patients (80% Women) treated With Radioactive Iodine, by Age at treatment and Estimated Stomach Dose

This supplementary material has been provided by the authors to give readers additional information about their work.

© 2019 Kitahara CM et al. *JAMA Internal Medicine*.

eTable 1. Relative Risks (RRs) and 95% Confidence Intervals (CIs) for Cancer-Specific Mortality at 100 mGy Organ/Tissue Absorbed Dose Among Hyperthyroid Patients Treated With RAI

Patient deaths occurring in the first five years after last RAI were excluded.

		**Dose-response**		
	Patients	Deaths	RR at 100 mGy (95% CI)[a]	P-value
**All solid cancers combined**				
Model 1[a]	16,322	1,984	1.06 (1.02, 1.10)	0.002
Model 2: Model 1, restricted to patients receiving a single RAI treatment	10,835	1,284	1.11 (1.02, 1.23)	0.008
Model 3: Model 1, restricted to patients receiving only RAI treatment (no surgery or anti-thyroid drugs)	6,197	759	1.14 (1.05, 1.27)	<0.001
**All solid cancers excluding female breast cancer**				
Model 1	16,322	1,693	1.05 (1.01, 1.10)	0.01
Model 2	10,835	1,103	1.13 (1.03, 1.27)	0.005
Model 3	6,197	655	1.11 (1.02, 1.24)	0.01
**Female breast cancer**				
Model 1	12,835	291	1.12 (1.003, 1.32)	0.04
Model 2	8,576	180	0.98 (<-0.97, 1.34)	>0.5
Model 3	4,850	104	1.05 (1.01, 1.18)	0.01

Abbreviations: CI, confidence interval; CLL, chronic lymphocytic leukemia; Gy, Gray; RAI, radioactive iodine
[a] Background rates include terms for sex, sex-specific attained age and birth cohort patterns, Graves' disease diagnosis (yes/no), additional treatment with surgery (yes/no), and additional treatment with anti-thyroid drugs (yes/no).

© 2019 Kitahara CM et al. *JAMA Internal Medicine*.

eTable 2. Estimates of Radiation-Associated Excess Solid Cancer Deaths (95% CIs) per 1,000 Hyperthyroidism Patients (80% Women) treated With Radioactive Iodine, by Age at treatment and Estimated Stomach Dose

	**Mean administered activity (MBq)**	**Radiation-associated excess solid cancer deaths (95% CI)**	
		Treatment at age 40	Treatment at age 50
**100 mGy stomach dose**	243 (±35): Graves' disease<br>236 (±38): Toxic nodular goiter		
Lifetime (all subsequent years after treatment)		13 (2, 27)	12 (2, 26)
First 20 years after treatment		1 (0.2, 3)	3 (0.4, 6)
**150 mGy stomach dose**	355 (±46): Graves' disease<br>353 (±47): Toxic nodular goiter		
Lifetime (all subsequent years after treatment)		19 (3, 40)	18 (3, 38)
First 20 years after treatment		2 (0.3, 4)	4 (0.6, 8)
**200 mGy stomach dose**	463 (±62): Graves' disease<br>464 (±53): Toxic nodular goiter		
Lifetime (all subsequent years after treatment)		25 (4, 53)	25 (4, 51)
First 20 years after treatment		2 (0.4, 5)	5 (1, 11)
**250 mGy stomach dose**	568 (±83): Graves' disease<br>568 (±92): Toxic nodular goiter		
Lifetime (all subsequent years after treatment)		32 (5, 66)	31 (5, 64)
First 20 years after treatment		3 (0.5, 7)	6 (1, 14)

© 2019 Kitahara CM et al. *JAMA Internal Medicine*.

© 2019 Kitahara CM et al. *JAMA Internal Medicine*.
